# The role of microRNA-155 in glomerular endothelial cell injury induced by high glucose

**DOI:** 10.1007/s11033-021-07106-1

**Published:** 2022-01-21

**Authors:** Kaiying He, Zhan Chen, Jing Zhao, Yang He, Rongrong Deng, Xin Fan, Jianqin Wang, Xiaochun Zhou

**Affiliations:** 1 Lanzhou University, Lanzhou, Gansu China; 2 Research Institute of Nephrology, Zhengzhou University, Zhengzhou, Henan China; 3 Department of Nephrology, Lanzhou University Second Hospital, No. 82, Cuiyingmen, Lanzhou, 730030 Gansu China

**Keywords:** Human renal glomerular endothelial cells, MiR-155, ETS-1, VCAM-1, MCP-1

## Abstract

**Objective:**

To investigate the role of microRNA-155-5p on apoptosis and inflammatory response in human renal glomerular endothelial cells (HRGEC) cultured with high glucose.

**Methods:**

The primary HRGEC were mainly studied, light microscopy was used to detect changes in cell morphology. Quantitative Real Time-Polymerase Chain Reaction, Western Blot, immunofluorescence were aimed to observe the mRNA and protein expression levels of target gene ETS-1, downstream factors VCAM-1, MCP-1 and cleaved caspase-3 in each group after high glucose treatment as well as transfection with miR-155 mimics or inhibitor.

**Results:**

The expression of inflammatory factors and apoptosis of HRGEC cells increased under high glucose treatment. Compared with normal-glucose treatment, the expression of microRNA-155 markedly increased in HRGECs treated with high-glucose, as well as the mRNA and protein levels of ETS-1, VCAM-1, MCP-1 and cleaved caspase-3. Overexpression of microRNA-155 remarkably downregulated mRNA and protein levels of ETS-1, VCAM-1, MCP-1 and cleaved caspase-3, whereas miRNA-155 knockdown upregulated their levels. In addition, HRGEC cells were transfected with miR-155 mimics and ETS-1 siRNA with high glucose stimulation. The expression of ETS-1 was positively correlated with the expression of downstream factors VCAM-1 and MCP-1. These results suggest that ETS-1 can mediate endothelial cell inflammation by regulating VCAM-1 and MCP-1.

**Conclusion:**

MiR-155 can negatively regulate the expression of target gene ETS-1 and its downstream factors VCAM-1, MCP-1 and cleaved caspase-3, thus mediating the inflammatory response and apoptosis of HRGEC.

**Supplementary Information:**

The online version contains supplementary material available at 10.1007/s11033-021-07106-1.

## Introduction

Diabetic nephropathy (DN) is a specific complication of long-term poor blood glucose control, which may lead to progressive renal function damage and cardiovascular risk [1, 2]. Nowadays, DN is the main cause of adult ESRD, which brings heavy burden to our country and society. Current studies indicate that the DN is caused by a combination of multiple factors, such as elevated blood glucose, changes in renal hemodynamics caused by hypertension, ethnic risk, genetic and metabolic interactions, etc. [3]. In recent years, the molecular level of DNA methylation, chromatin histone modification, new transcripts and functional non-coding RNAs, for example, microRNA and long non-coding RNA have been rapidly studied in the fields of glomerular immune inflammatory response, epithelial mesenchymal transformation, cell apoptosis, mitochondrial damage, podocyte endothelial cell interaction, etc. [4–7]. However, there is still no conclusion on the occurrence and progress of DN. Therefore, it is urgent to further study the pathogenesis of DN and find more specific biomarkers and targets to assist in the early diagnosis and treatment of DN at the gene level [8, 9]. This study selected the primary human glomerular endothelial cells as the research object, because it is located in the glomerular capillary the innermost, and it is vulnerable to the blood sugar, blood lipids, the stimulation of inflammation factors such as damage [10]. Injury of endothelial cells lead to abnormal secretion of related cytokines acting on adjacent potocytes and mesangial cells, leading to a vicious cycle, which result in increased production of extracellular matrix, thickening of basement membrane, and interstitial fibrosis aggravating glomerulosclerosis [11–13]. Therefore, the in-depth study on the injury mechanism of glomerular endothelial cells under high glucose treatment is of great significance for finding new targets.

MicroRNAs are small endogenous non-coding RNA with a length of about 20 bp, which bind to the 3 'untranslated region of mRNA of target genes through base complementary pairing, thereby degrading mRNA or inhibiting its translation and regulating downstream protein expression. Multiple studies have shown that microRNA plays an important role in cell proliferation, apoptosis, inflammation, accumulation of extracellular matrix, glomerular fibrosis and the occurrence and development of diabetes [14–20]. MicroRNA, as a new biomarker, has been widely studied and plays an important role in inducing injury of endothelial cells and podocyte as well as the occurrence and development of diabetic nephropathy. It has high potential application value in the early diagnosis and treatment of DN. Previous studies have shown that miR-155 as a multifunctional miRNA, plays an important role in cell differentiation and proliferation, inflammation, apoptosis and autophagy [21–25]. Huang et al. [26] found that miR-155 expression was increased in renal tissues of DN patients, and mainly expressed in glomerular vascular endothelial cells, mesangial cells and renal tubule interstitium. The results of luciferase reporter gene showed that E26 transformation-specific Sequence 1 (ETS-1) may be a potential target gene of miR-155. Further detection of serum miRNAs in diabetic patients showed abnormal expression of miR-155 in diabetic patients compared with healthy controls, and the expression of miR-155 was significantly different in microproteinuria and macroproteinuria groups, and was positively correlated with eGFR in DN patients and negatively correlated with urinary protein excretion rate.

Based on the current theoretical understanding and previous research of our group, we speculated that circulating miR-155 may be involved in the occurrence of DN by regulating ETS-1 and is related to the injury of glomerular endothelial cells in diabetic nephropathy. As the mechanism of miR-155 in glomerular endothelial cell injury induced by high glucose is poorly understood, our study intends to construct a transfection model of human glomerular endothelial cells with miR-155 mimics/inhibitor under high glucose treatment. In order to further understand the role of miR-155 in diabetic glomerular endothelial injury, and initially clarify its mechanism. It provides experimental basis for clinical early warning and early diagnosis of diabetic nephropathy, as well as finding new targets for intervention, and it has important scientific value and clinical significance for the prevention and treatment of diabetic nephropathy.

## Materials and methods

### Cells

The primary human renal glomerular endothelial cells (HRGEC) used in this study were all purchased from ScienCell Research Laboratory (ART. 4000, Lot. 15373). Cells were cultured in precoated flask with fibrin coating solution with high-glucose (30 mmol/L) or normal-glucose Endothelial Cell Medium (ECM, ScienCell, USA) containing 5% serum, 1% growth factor, 1% Penicillin–Streptomycin solution, and placed in an incubator with 5% CO_2_ at 37 ℃. When the cell culture reached about 90% fusion, the passage was carried out.

### miRNA transfection

In 25T flask, according to the instructions of riboFECTTMCP transfection reagent, 50 nM miRNA-155 mimics and mimics negative control, 100 nM inhibitor and inhibitor negative control were transfected into HRGEC cells respectively, and the transfected cells were obtained at specified time to detect cell apoptosis, expression of inflammatory factors and miR-155 levels. Sequences of miR-155 mimic, mimic negative control, miR-155 inhibitor, inhibitor control are shown in Supplementary Table 1.

### QRT-PCR

Total RNA was extracted by Trizol, after RNA concentration was measured, cDNA synthesis was performed using reverse transcription kit (TakaRa, Otsu, Shiga, Japan) and the Ct values for miR-155, mRNA were normalized to U6, β-actin respectively. The relative expression levels of miRNA-155, ETS-1, VCAM-1, MCP-1 and cleaved caspase-3 were calculated by 2^−∆∆CT^ method. The primer sequences are shown in Supplementary Table 2.

### Western blot

The cells were lysed with protein lysis buffer on ice for 30 min. Then the protein sample was quantified by bicinchoninic acid (BCA, Biosharp, Hefei, China), and the loading buffer was added and heated for denaturation. The protein samples were subjected to electrophoresis, electrortansfer, blocking, and incubation with primary and secondary antibodies. The absorbance of the protein bands was scanned and analyzed in a gel image analysis system using enhanced chemiluminescence (ECL, Millipore, USA) system.

### Cell immunofluorescence

Cell slipper in 24-well plate was cultured in 37 ℃ sterile CO_2_ incubator until 80% cells were fused, and different treatments were carried out for each experimental group according to the experiment. After 48–72 h induction, the cells were fixed with 4% PFA for 40 min, then washed with DPBS, 0.3% TritonX-100 permeable cells for 30 min, and 3% BSA blocked for 20 min. Fluorescent primary antibody was incubated at 4 ℃ for 12 h, and fluorescent secondary antibody was incubated at room temperature for 2 h, then washed with DPBS. The images were taken under confocal laser microscope after dye nuclear with DAPI and sealing.

### Statistical analysis

Statistical Program for Social Sciences (SPSS) 20.0 software was used for Statistical analysis of the data in this paper. The measurement data were expressed as mean ± standard deviation (X ± S). Two independent sample T tests were used to compare the two groups. Univariate ANOVA was used to compare the groups. The Least Significant Difference (LSD-T) test was used for pairwise comparison between multiple groups. P ≤ 0.05 was considered as significant and statistically significant.

## Results

### Light microscope results of cell growth condition after normal glucose, hyperosmolar, high glucose and transfection with miR-155

**Fig. 1:**
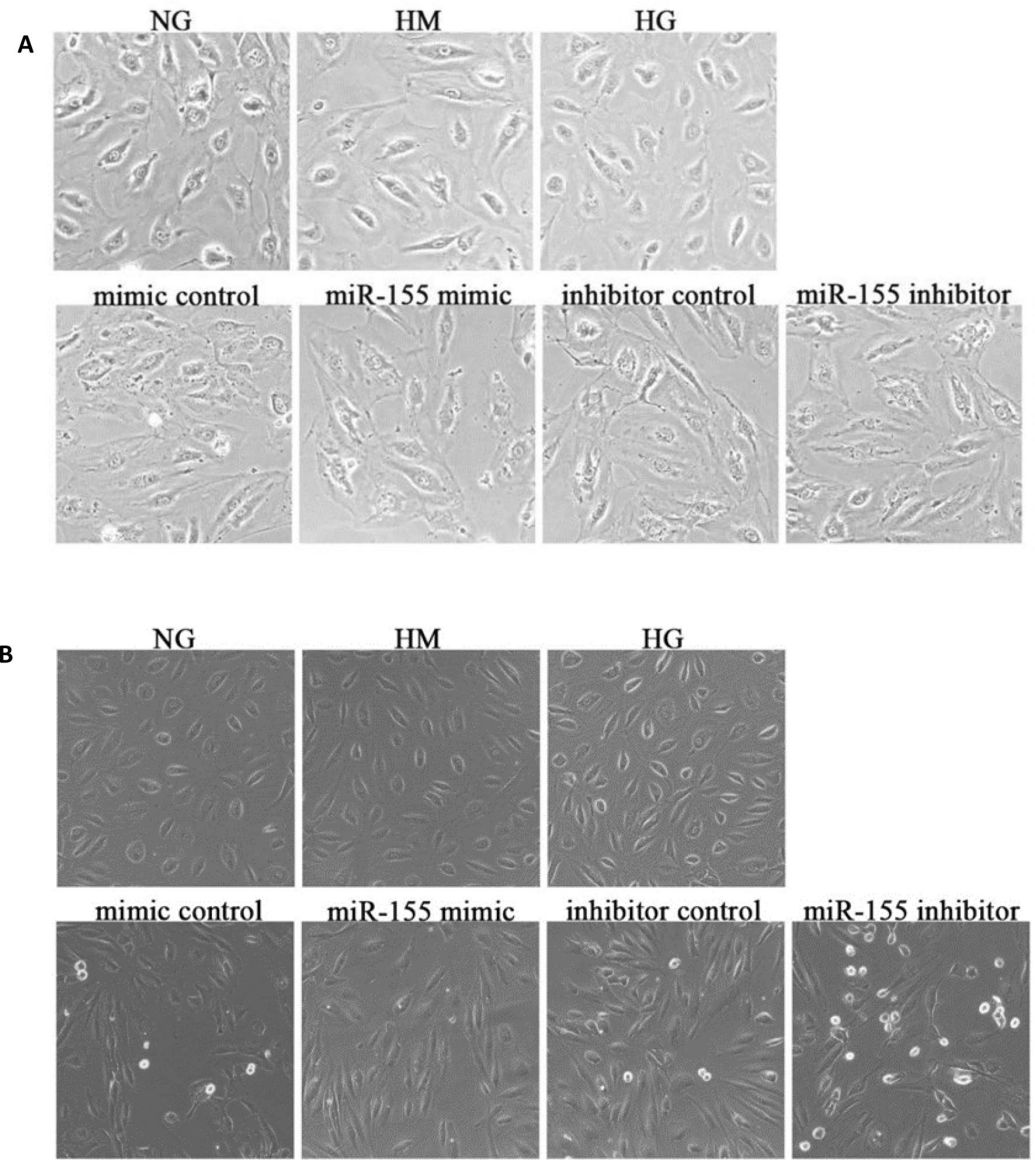
**A** Light microscope results of HRGECs growth under different conditions. **B** Light microscope results of apoptosis of HRGECs under different conditions. *NG* normal glucose group, *HM* hypertonic group, *HG* high glucose group, *mimic control* high glucose + mimic negative control, *miR-155 mimic* high glucose + miR-155 mimic, *Inhibitor control* high glucose + inhibitor negative control, *miR-155 inhibitor* high glucose + miR-155 inhibitor (×20)

In this study, endothelial cell damage induced by high glucose was investigated. In order to simulate the high glucose or high osmotic environment, the concentration of D-glucose or mannitol was added to ECM medium to reach 30 mmol/L, miR-155 was transfected after high-glucose stimulation. After 48 h of stimulation, cells in each group were changed with fresh culture medium to observe and photograph under light microscope. It was found that cells in the normal glucose (NG), high mannitol (HM) and high glucose (HG) groups had good growth state, full morphology and tight intercellular connections. The cell density could reach 100% after 3 days of subculture. Compared with NG, HM and HG groups, cell density in the transfection group was reduced, cell membranes were folded, granular substances appeared in cytoplasm and nucleus, and cell morphology was changed (Fig. 1A). In addition, compared with HM, HG and transfection control group, a large number of intact and adherent round bubble cells were found in miR-155 inhibitor group, but no such cells were found in HM and HG group, and few cells in miR-155 mimic group. It is speculated that these bubble cells may be vacuolated changes of cells in the early stage of apoptosis (Fig. 1B).

### MiRNA-155 expression increased in HRGECs in high glucose treatment

**Fig. 2:**
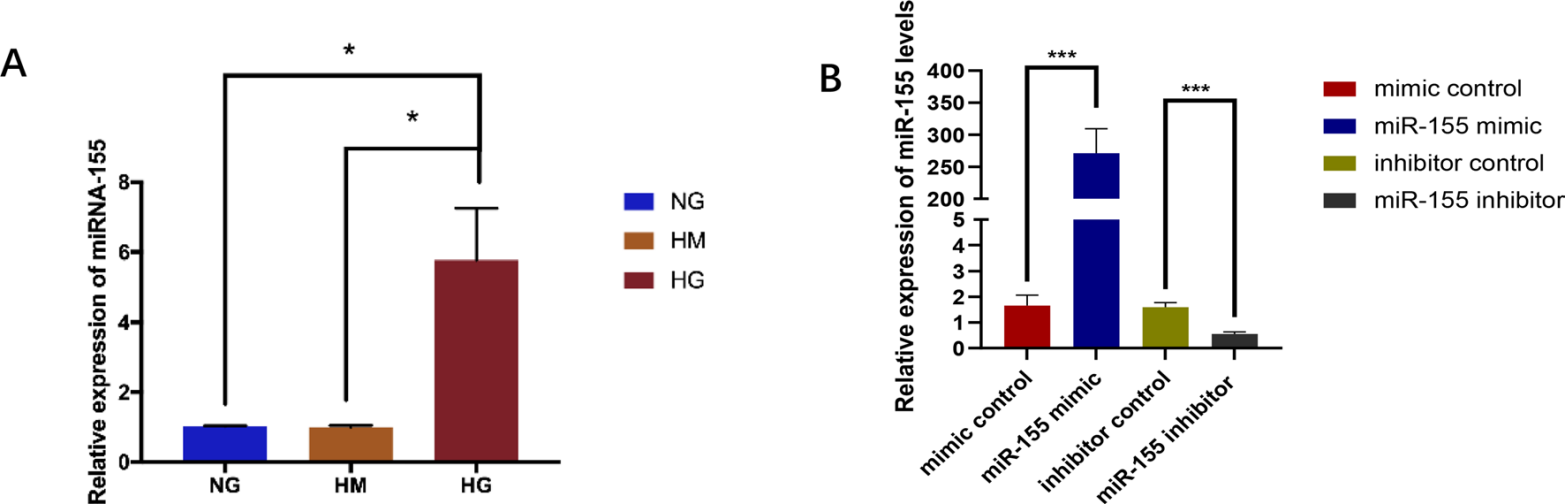
**A** The relative expression level of miRNA-155 in HRGEC cells after corresponding treatment. **B** Expression levels of miR-155 after transfection with miR-155 mimic, mimic control, miR-155 inhibitor and inhibitor control. *NG* normal sugar group, *HM* hypertonic group, *HG* high glucose group, *mimic control* mimic negative control, *Inhibitor control* inhibitor negative control. *P ≤ 0.05, ***P < 0.001

After high glucose, high osmotic and normal glucose stimulation for 24 h, the total RNA was extracted, respectively. QRT-PCR was used to verify the expression of miRNA-155 under different treatment. As qRT-PCR data revealed, microRNA-155 was highly expressed in HG group relative to NG group and HM group (P ≤ 0. 05, Fig. 2A). The expression level of miRNA-155 in HRGEC cells after transfection.

HRGEC cells were transfected with miR-155 mimic control, miR-155 mimics, miR-155 inhibitor control and miR-155 inhibitor respectively. QRT-PCR was used to verify the transfection efficiency. The results of qRT-PCR showed that the expression of miR-155 in miR-155 mimic group was significantly higher than that in the control group (P ≤ 0. 05), in addition, the expression of miR-155 in miR-155 inhibitor group is markedly decreased compared with the control group (P ≤ 0. 05, Fig. 2B).

### HG induction promoted inflammation and apoptosis of HRGEC cells

**Fig. 3:**
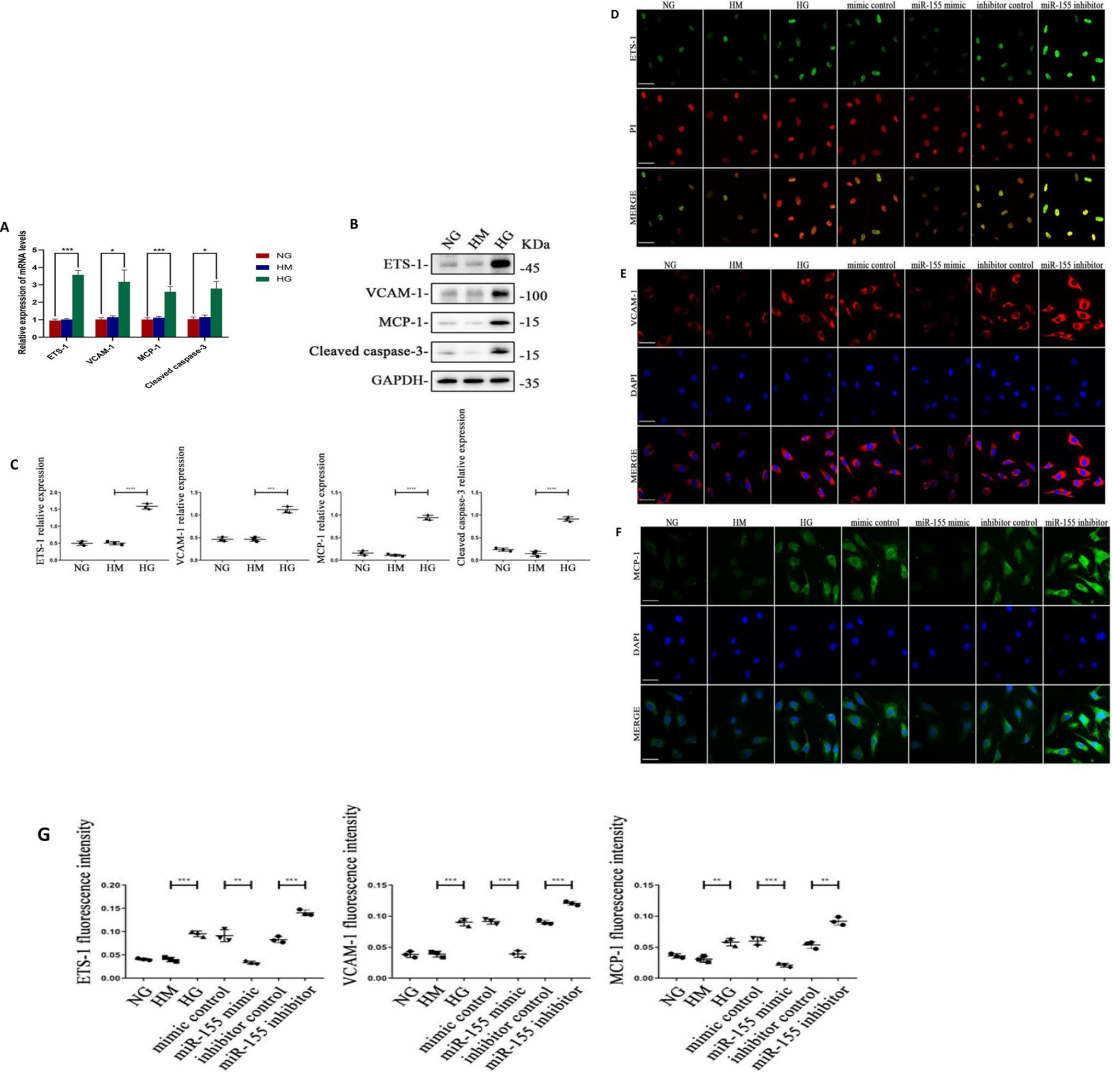
**A** The mRNA expression levels of ETS-1, VCAM-1, MCP-1, Cleaved caspase-3 in HRGEC after corresponding treatment. **B**, **C** The protein expression levels of and quantitative analysis of the relative expression levels of ETS-1, VCAM-1, MCP-1, Cleaved caspase-3 in HRGEC stimulated by high glucose. **D**–**G** Immunofluorescence of ETS-1, VCAM-1, MCP- 1 and quantitative immunofluorescence analysis of ETS-1, VCAM-1 and MCP-1 in HRGEC with high glucose or high glucose + miR-155 transfection. *NG* normal glucose group, *HM* hypertonic group, *HG* high glucose group, *mimic control* high glucose + mimic negative control, *miR-155 mimic* high glucose + miR-155 mimic, *Inhibitor control* high glucose + inhibitor negative control, *miR-155 inhibitor* high glucose + miR-155 inhibitor. bar = 50 μm. *P < 0.05, **P < 0.01, ***P < 0.001

The HRGEC cells were cultured with high glucose, high osmotic or normal glucose, meanwhile the RNA and the protein were extracted after 24 h, 48 h respectively. QRT-PCR results showed that compared with NG and HM groups, the mRNA levels of E26 transformation-specific Sequence 1 (ETS-1), Vascular Cell Adhesion Molecule 1 (VCAM-1), Monocyte Chemotactic Protein 1 (MCP-1) and apoptosis related Protein, such as Cleaved caspase-3 were greatly increased, which was statistically significant (P ≤ 0.05, Fig. 3A). Western blotting and quantitative analysis showed that ETS-1, VCAM-1, MCP-1 and Cleaved caspase-3 were significantly upregulated in the HG group compared with NG and HM groups, with statistical significance (P ≤ 0.05), while there was no significant difference between NG and HM groups (Fig. 3B, C). The results of immunofluorescence showed that ETS-1 was expressed in the nucleus, VCAM-1 and MCP-1 in the cytoplasm. After HGERC cells were stimulated by high glucose, the fluorescence of ETS-1, VCAM-1 and MCP-1 was enhanced, and the results were consistent with western-blot results (Fig. 3D–G).

### MiR-155 overexpression inhibited the expression of ETS-1, VCAM-1, MCP-1 and cleaved caspase-3 under high glucose treatment

**Fig. 4:**
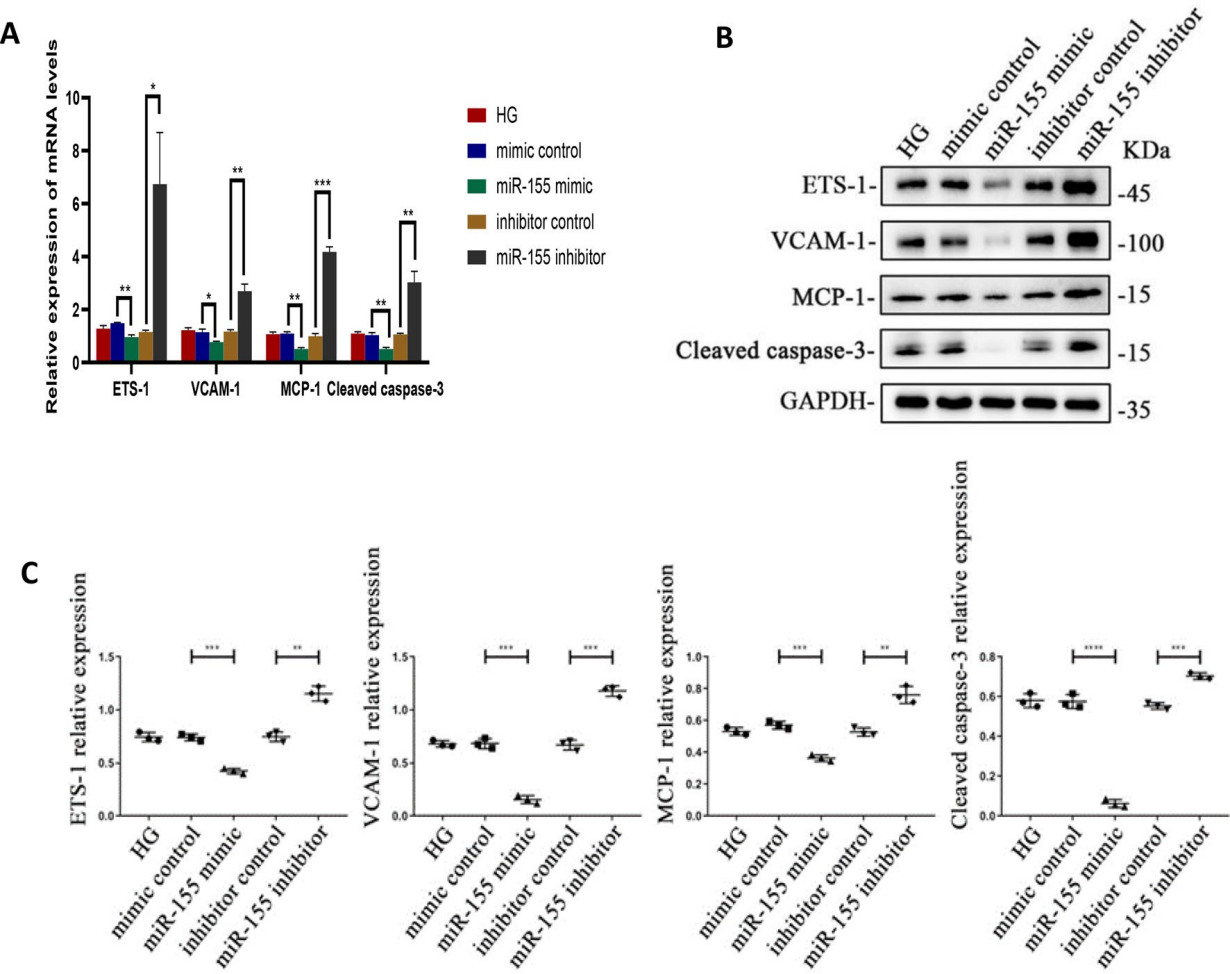
**A** The mRNA expression levels of ETS-1, VCAM-1, MCP-1 and cleaved caspase-3 in HRGEC with high glucose treatment after miR-155 transfection. **B**, **C** The protein expression level and quantitative analysis of ETS-1, VCAM-1, MCP-1 and cleaved caspase-3 after transfection of miR-155 in HRGEC with high glucose treatment. *HG* high glucose control group, *mimic control* high glucose + mimic negative control, *miR-155 mimic* high glucose + miR-155 mimic, *Inhibitor control* high glucose + inhibitor negative control, *miR-155 inhibitor* high glucose + miR-155 inhibitor. *P < 0.05, **P < 0.01, ***P < 0.001

In order to determine the effects of miR-155 on ETS-1, VCAM-1, MCP-1 and cleaved caspase-3, HRGECs were transfected with miR-155 mimics, miR-155 inhibitor, mimic control and inhibitor control under high glucose treatment. The corresponding RNA and protein were extracted 24 h and 48 h after transfection respectively. QRT-PCR results showed that the mRNA levels of ETS-1, VCAM-1, MCP-1 and Cleaved caspase-3 in miR-155 mimic group were decreased compared with the control group, meanwhile, the mRNA levels of those mRNA increased in miR-155 inhibitor group, which was statistically significant (P ≤ 0.05, Fig. 4A). Western-blot and quantitative analysis showed that the protein expression levels of ETS-1, VCAM-1, MCP-1 and Cleaved caspase-3 in miR-155 mimic and miR-155 inhibitor group were significantly different compared with the control group (P ≤ 0.05). There was no significant difference between mimic control and inhibitor control group and high glucose control group (Fig. 4B, C). The results of cell immunofluorescence and quantitative analysis were consistent with those of Western-blot (Fig. 3D–G).

### Regulation of downstream effector factors by inhibition of ETS-1 in high glucose treatment

**Fig. 5:**
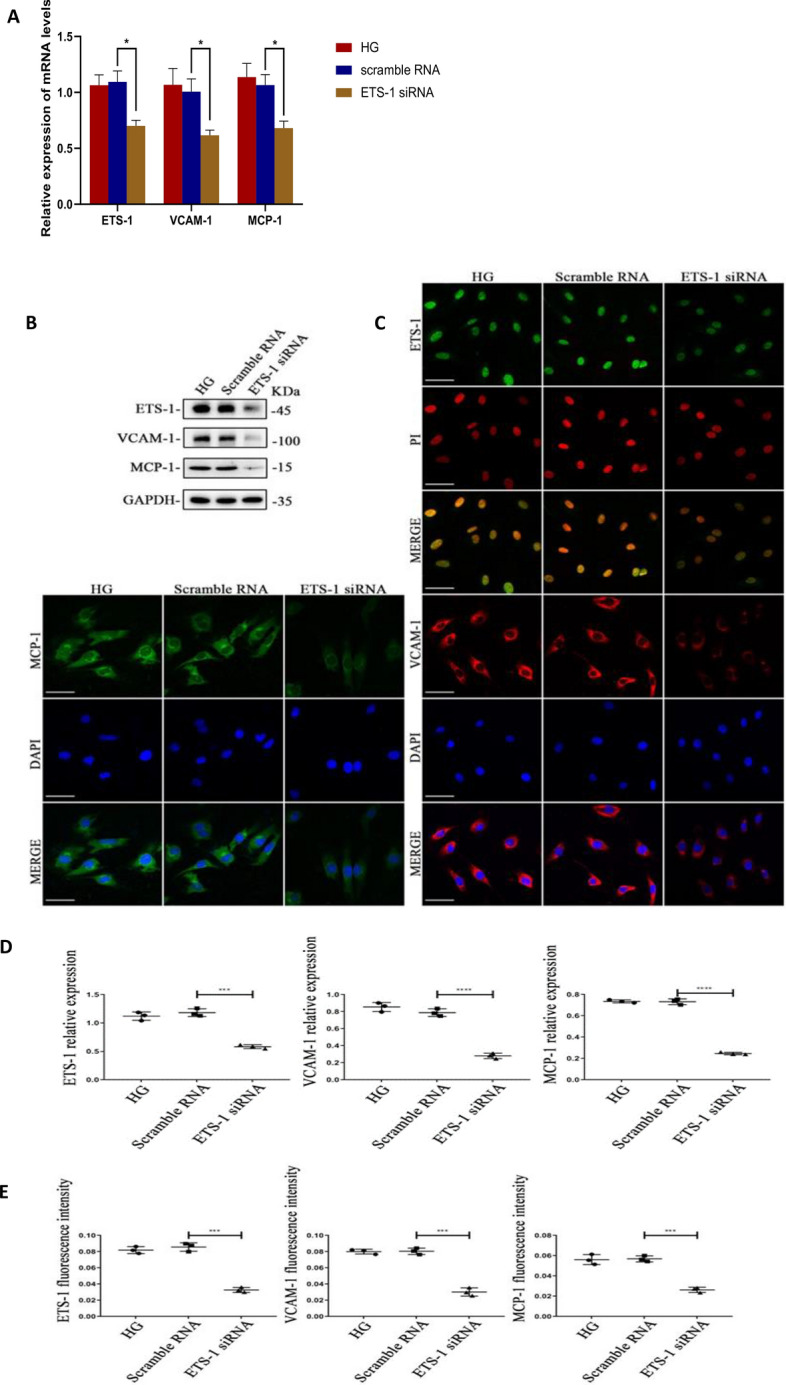
**A** The mRNA levels of ETS-1,VCAM-1 and MCP-1 in HRGEC with high glucose and high glucose + ETS-1 siRNA. **B**–**E**, The protein levels and quantitative analysis of ETS-1, VCAM- 1, MCP-1 in HRGEC with high glucose and high glucose + ETS-1 siRNA. *HG* high glucose group, *Scramble RNA* high glucose + scramble RNA, *ETS-1 siRNA* high glucose + ETS-1 siRNA. Bar = 50 μm. *P < 0.05, ***P < 0.001

In order to further clarify the regulation of ETS-1 on downstream effector factors VCAM-1 and MCP-1, ETS-1 siRNA and scramble RNA were transfected into HRGECs with high-glucose treatment. QRT-PCR results showed that the mRNA levels of ETS-1, VCAM-1 and MCP-1 in the ETS-1 siRNA group were significantly decreased compared with the control group, with statistical significance (P ≤ 0.05), and there was no significant difference between the scramble RNA group and the HG group (Fig. 5A). Western-blot and quantitative analysis showed that the protein expressions of ETS-1, VCAM-1 and MCP-1 in the ETS-1 siRNA group were significantly decreased compared with the control group, with statistically significant differences (P ≤ 0.05), while there was no significant difference between the scramble RNA group and the HG group (Fig. 5B, D). The results of cell immunofluorescence and quantitative analysis were consistent with those of Western-blot (Fig. 5C, E).

## Discussion

MiR-155 is a typical multifunctional miRNA, which plays a crucial role in the regulation of numerous cells and blood vessels. However, its mechanism on HRGECs dysfunction has not been fully clarified. Existing studies have shown that miR-155 is involved in cell differentiation, proliferation, inflammation, immunity, tumorigenesis, autophagy, apoptosis, and the development regulation of a variety of biological tissues [21–25]. In this study, HRGECs stimulated by high glucose were selected to simulate the internal environment during the occurrence of diabetes mellitus, and the regulation of miR-155 on ETS-1 and downstream factors was studied by transfected endothelial cells with miR-155 mimics or inhibitor.

At present, the specific target gene of miR-155 is not clear. The results of online gene prediction software TargetScan and double luciferase reporter gene in the early stage of our research group indicate that ETS-1 may be the target gene related to miR-155 in DN. ETS-1 (E26 transformation-specific Sequence 1) belongs to the E26 transformation-specific transcription factor superfamily. As a proto-oncogene, it can be upregulated by angiotensin II (Ang II) in the vascular system and glomerulus to promote inflammatory response [27, 28]. In addition, it plays an important role in regulating energy metabolism, vascular development and generation, vascular inflammation and remodeling of cancer cells [29, 30]. Its target genes include receptor tyrosine kinases, MMPs and intercellular adhesion molecules. As an endothelial adhesion receptor, VCAM-1 plays an important role in leukocyte recruitment in cellular immune response, at the same time, it can promote the adhesion of leukocytes to vascular wall and induce inflammation. It is also associated with tumor growth, metastasis and angiogenesis [31]. MCP-1 is a member of the CC subfamily of chemokines, which can chemotactic monocytes, T lymphocytes, macrophages, and affect their phagocytosis as well as antibody production [32]. IL-1 induces MCP-1 production in glomerular cells during renal inflammation. In hyperlipidemia, LDL binds to glomerular cells to stimulate their production of MCP-1 and chemotactic monocytes. Zhu et al. [33] showed that ETS-1 and its downstream factors FLT-1, MCP-1 and VCAM-1 were up-regulated after Ang II stimulated umbilical vein endothelial cells, while overexpression of miR-155 and miR-221 partially reversed this effect. Therefore, we believe that ETS-1, VCAM-1, MCP-1 may play an important role in inflammatory response, angiogenesis and extracellular matrix deposition during the development of DN.

Therefore, different methods were adopted in this study to verify the regulation of miR-155 on ETS-1 and downstream factors VCAM-1, MCP-1 by overexpression or inhibition of miR-155 on HRGECs cultured with high glucose. It was found that after high glucose stimulated endothelial cells, QPCR, Western blotting, and immunofluorescence results showed that the expressions of ETS-1, VCAM-1, MCP-1, and cleaved caspase-3 were increased, further indicating that high glucose can induce endothelial cell inflammation and apoptosis. After transfection with miR-155 mimics or inhibitor, Western blotting and immunofluorescence showed that the expression of ETS-1 decreased when miR-155 was overexpressed, while increased when miR-155 was inhibited. The targeted regulation of miR-155 on ETS-1 was preliminatively verified. Meanwhile, VCAM-1, MCP-1, Cleaved caspase-3 protein expression showed the same trend as ETS-1. To further verify the regulation of ETS-1 on downstream factors VCAM-1 and MCP-1, ETS-1 siRNA was transfected into HRGECs. The results showed that the expression of ETS-1 and its downstream factors (VCAM-1, MCP-1) were down-regulated in the ETS-1 siRNA group and miR-155 mimic group. Therefore, miR-155 can negatively regulate the expression of ETS-1 and then act on MCP-1, VCAM-1 to participate in the inflammatory response of HG treatment. In terms of cell apoptosis, Liao et al. [34] reported that miR-155 is up-regulated in hepatocellular carcinoma, and the increased miR-155 can target FoxO3a expression and then inhibit bcl-2, caspase-3 and caspase-9 activities, thus inhibiting cell apoptosis and promoting cell proliferation in patients with hepatocellular carcinoma. Xu et al. [35] found that down-regulation of miR-155 can significantly inhibit proliferation, migration, invasion and promote apoptosis of psoriatic cells through the PTEN signaling pathway. In this study, mir-155 overexpression in endothelial cells could inhibit cleaved caspase-3 expression and thus inhibit apoptosis of endothelial cells.

In addition, there are still some limitations and deficiencies in this study. Firstly, only one target gene of miR-155 was involved in this study, while miRNAs simultaneously regulate multiple target genes in vivo. The expression of miR-155 and its target genes have not been verified in animal models or human kidney tissues and there is a lack of miR-155 overexpression or knockout mouse model to clarify the regulatory effect of miR-155 in animals. Secondly, classical flow cytometry and TUNEL analysis were not used to explain the regulatory effect of miR-155 on endothelial cell apoptosis. Therefore, it is still necessary to further study the relationship between miR-155 and the development of DN, so as to provide new ideas for basic and clinical research about DN.

Combined with relevant reports, this study initially concluded that miR-155 may mediate HRGECs injury through negative regulation of target gene ETS-1 and downstream factors VCAM-1 and MCP-1. Meanwhile, miR-155 can negatively regulate the expression of apoptosis-related protein Cleaved caspase-3 and participate in endothelial cell apoptosis, so miR-155 is expected to serve as a marker of endothelial cell injury in diabetic nephropathy. At present, this study is relatively limited, and animal experiments and large-sample clinical studies are still needed as support to further reveal the role of miR-155 in the development of DN and provide a new path for the early diagnosis and treatment of DN.

## Supplementary Information

Below is the link to the electronic supplementary material.

Supplementary file1 (DOCX 15 kb)

## Data Availability

All data generated or analysed during this study are included in this article. Further enquiries can be directed to the corresponding author.
