# Supplementary material for: The role of microRNA-155 in glomerular endothelial cell injury induced by high glucose
Source: Mol Biol Rep. 2022 Jan 21;49(4):2915–24. doi: 10.1007/s11033-021-07106-1 (PMC8924107; doi:10.1007/s11033-021-07106-1)
Supplement: Supplementary file 1 — Supplementary file1 (DOCX 15 kb) [file 11033_2021_7106_MOESM1_ESM.docx]

Supplementary table 1 Sequences of miR-155 mimic/inhibitor/mimic NC/inhibitor NC for transfection

| hsa-miR-155-5p mimic | 5'-UUAAUGCUAAUCGUGAUAGGGGUU-3' |
| --- | --- |
| hsa-miR-155-5p inhibitor | 5'-AACCCCUAUCACGAUUAGCAUUAA-3' |
| mimic NC | 5'-UUUGUACUACACAAAAGUACUG-3' |
| inhibitor NC | 5'-CAGUACUUUUGUGUAGUACAAA-3' |

Supplementary table2 Sequences of primers for qRT-PCR

| Gene | Sequences |  |
| --- | --- | --- |
| hsa-ETS-1 | Sense | 5’-GAAGGATGGGCAAATCTGGTC-3’ |
|  | Antisense | 5’-GAATGGAGAAGGGAACAAAAGTGA-3’ |
| hsa-VCAM-1 | Sense | 5’-TCGTGAAACATCTCCGTACCA -3’ |
|  | Antisense | 5’-TAATAAGCAAAGGGAGCACTGG-3’ |
| hsa-MCP-1 | Sense | 5’-GCAATCAATGCCCCAGTCA  -3’ |
|  | Antisense | 5’-ACACTTGCTGCTGGTGATTCTT -3’ |
| hsa-Cleaved caspase-3 | Sense | 5’-TACTCCTTCCATCAAATAGAACCAC -3’ |
|  | Antisense | 5’-TCATAATGACTGCACCAGTATCCA  -3’ |
| hsa-GAPDH | Sense | 5’-CCAGCAAGAGCACAAGAGGA  -3’ |
|  | Antisense | 5’-GTCTACATGGCAACTGTGAGGAG  -3’ |
